# Curcumin and Quercetin Ameliorated Cypermethrin and Deltamethrin-Induced Reproductive System Impairment in Male Wistar Rats by Upregulating The Activity of Pituitary-Gonadal Hormones and Steroidogenic Enzymes 

**DOI:** 10.22074/ijfs.2018.5160

**Published:** 2018-01-15

**Authors:** Poonam Sharma, Irshad Aslam Khan, Rambir Singh

**Affiliations:** 1 Department of Zoology, Indira Gandhi National Tribal University, Amarkantak, Madhya Pradesh, India; 2 Department of Zoology, Bundelkhand University, Jhansi, Uttar Pradesh, India; 3 Department of Biomedical Sciences, Bundelkhand University, Jhansi, Uttar Pradesh, India

**Keywords:** Curcumin, Cypermethrin, Deltamethrin, Quercetin

## Abstract

**Background:**

Dietary antioxidants protect tissues and organs against insecticides/xenobiotic-induced damage. In the present study, we evaluated the results of exposure to synthetic pyrethroid insecticides, cypermethrin (Cyp) and deltamethrin (Del) and possible protective effects of curcumin and quercetin on reproductive system in male Wistar rats.

**Materials and Methods:**

In this controlled experimental study, 42 male Wistar rats were randomly divided into 7 groups of 6 animals. Group A served as control, group B was exposed to Cyp (2 mg/kg.bw), group C was exposed to Del (2 mg/kg.bw), group D was exposed to Cyp+Del (2 mg/kg.bw each), group E was exposed to Cyp+Del and treated with curcumin (100 mg/kg.bw), group F was exposed to Cyp+Del and treated with quercetin (100 mg/kg.bw) and group G was exposed to Cyp+Del and treated with quercetin+curcumin for 45 days.

**Results:**

Exposure to Cyp and Del caused decreases in reproductive organs weight, sperm count, sperm motility, level of sex hormones viz. testosterone (T), follicle stimulating hormone (FSH) and luteinizing hormone (LH), steroidogenic enzymes viz. 3β-hydroxyl steroid dehydrogenase (3β-HSD) and 17β-HSD, non-enzymatic antioxi- dant glutathione (GSH) and enzymatic antioxidants viz. superoxide dismutase (SOD), catalase (CAT), glutathione peroxidase (GPx), glutathione-S-transferase (GST) and glutathione reductase (GR) activity and increases in sperm abnormalities and lipid peroxidation (LPO). The exposure also adversely affected the histo-achitecture of testes. Single and combined treatment with curcumin and quercetin significantly ameliorated Cyp and Del-induced damage in reproductive system.

**Conclusion:**

Curcumin and quercetin protected against Cyp and Del-induced reproductive system toxicity and oxidative damage in rats. The increases in activities of 3β-HSD and 17β-HSD with concomitant increases in testosterone were mainly responsible for ameliorating effects of curcumin and quercetin. Curcumin showed slightly better activity as compared to quercetin. The combination of both antioxidants offered more protection compared to each one alone.

## Introduction

Synthetic pyrethroids insecticides are widely used because of their high effectiveness against a large number of insects, rapid biodegradation, low mammalian toxicity and target-oriented mechanism of action (1). Cypermethrin (Cyp) and deltamethrin (Del) are synthetic pyrethroids used in agriculture, veterinary and public health programs for management of insects and pests (2). Although pyrethroids are considered to be safe for humans, indiscriminate uses of these insecticides have induced carcinogenicity, neurotoxicity, genotoxicity and developmental toxicity in domestic animals and humans (3). The reproductive toxicity of Cyp (4) and Del (5) was previously evaluated in our laboratory. Increased oxidative stress and augmented generation of reactive oxygen species (ROS) are among the underlying mechanisms via which these insecticides induced toxicity.

Dietary antioxidants, chiefly plant phenolics, flavonoids and carotenoids that have ROS scavenging activity, are considered important for a healthy life. Curcumin, a polyphenolic compound obtained from turmeric is an excellent antioxidant and possesses a number of pharmacological activities (6). Quercetin, the flavonoid present in several vegetables and fruits also possesses antioxidant and other biological activities (7). The ameliorative effects of curcumin (8) and quercetin (9) on xenobiotic-induced reproductive toxicity have largely been attributed to their ability in decreasing oxidative stress in testicular tissue, in laboratory animals.

The present study was planned to investigate the role of curcumin and quercetin in Del and Cyp-induced reproductive toxicity in male Wistar rats. Apart from evaluating their antioxidant potential, we explored the effect of these phytochemicals on sperm parameters, hormones of the pituitary-gonadal axis and enzymes involved in testosterone biosynthesis.

## Materials and Methods

Male Wistar rats, weighing about 200-250 g, were used in this controlled experimental study. Animals were kept in the animal house at 22 ± 3°C, with relative humidity of 45-55%, and 12 hours/12 hours dark/light cycles. The animals were fed with pelleted diet and water ad-libitum. All animal experiments were performed as per approval of the Institutional Animal Ethics Committee (BU/Pharma/ IAEC/12/032).

### Chemicals

Technical grade Cyp (99.2%) and Del (98.5%) were obtained from Gharda chemicals (Mumbai, India). Curcumin (95%) was purchased from Sigma-Aldrich (St. Louis, MO. USA) and quercetin dihydrate (98%) from Himedia (Mumbai, India). All other chemicals used in this study were of high purity and purchased from standard firms.

### Treatment schedule

Forty two male Wistar rats were randomly divided into 7 groups of 6 animals. Cyp, Del, curcumin and quercetin were dissolved in polyethylene glycol (10) and administered orally for 45 days. The doses of curcumin (11), quercetin (12) and Del (13) were selected based on previous studies. For effective comparison, equivalent doses of Cyp and Del were used. Group A served as control and each animal in the group received 1 ml of polyethylene glycol. Group B was exposed to Cyp (2 mg/kg.bw), group C was exposed to Del (2 mg/ kg.bw), group D was exposed to Cyp+Del (2 mg/kg.bw each), group E was exposed to Cyp+Del and treated with curcumin (100 mg/kg.bw), group F was exposed to Cyp+Del and treated with quercetin (100 mg/kg.bw) and group G was exposed to Cyp+Del and treated with quercetin (100 mg/ kg.bw)+curcumin (100 mg/kg.bw) for 45 days.

At the end of the experiment, rats were sacrificed by cervical dislocation, under ketamine-induced anesthesia. The testes and epididymis were removed and weighted. The epididymis was used for sperm motility and sperm morphology studies. One testis was used for sperm head counts and the other was used for estimation of lipid peroxidation, enzymatic and non-enzymatic antioxidants and steroidogenic enzymes. A part of testis was kept in 10% formaldehyde for histological studies. Blood was taken from the heart and used for estimation of various reproductive hormones.

### Estimation of sperm parameters

#### Sperm head counts

Sperm head counts was performed using a hemocytometer as described by Choi et al. (14). The testis was dissected and tunica albuginea (outer covering) was removed. The testis was minced in a solution consisting of 0.9% NaCl and 0.05 triton X and homogenized for 2 minutes at highest speed using a tissue homogenizer. Testis homogenate (10-15 µl) was placed on hemocytometer and after 5 minutes, sperm heads were counted in red blood corpuscles (RBC) chamber at ×40 magnification.

#### Sperm motility

A segment of distal cauda epididymis was removed and kept in 2 ml of Dulbecco’s phosphate-buffered saline (PBS), maintained at 36-38°C on a water bath. Cauda was minced sufficiently to disperse the sperm for 1-5 minutes and gently mixed using pasture pipette. The test sample (5-10 µl) was loaded into the hemocytometer chamber and the motile sperms were counted in white blood cells (WBC) counting area. Sperms were counted as motile if they exhibited any type of movement/motion. Hemocytometer was placed on ice for 10-20 seconds to render all the sperms immotile for counting the total sperms (15).

#### Sperm morphology

Cauda epididymis was minced with the help of a razor, in 1 ml of 0.9% saline and 1 ml of 10% neutral buffered formaldehyde was added. The suspension was diluted with water to a volume suitable for performing the assay. Next, 1-2 ml of 1% Eosin was added to 20 ml of the above-mentioned mixture and incubated at room temperature for 45 to 60 minutes. One drop of this suspension was taken on slide and a smear was prepared for studying sperm morphology. The head and tail abnormalities were expressed as percentage.

### Biochemical estimations

#### Estimation of testosterone, follicle stimulating hormone and luteinizing hormone

At the end of the experiment, blood was taken from the heart and centrifuged and serum was separated for the estimation of reproductive hormones. Testosterone (T), follicle stimulating hormone (FSH) and luteinizing hormone (LH) levels were estimated using rat specific ELISA kits (Qayee-Bio Life Science, China).

#### Estimation of steroidogenic enzymes

Testis tissue (100 mg) was rinsed and homogenized in 1 ml of 1X PBS and stored at -20°C, overnight. After two freeze-tha wcycles to break the cell membranes, the homogenate was centrifuged at 5000 g for five minutes in a refrigerated centrifuge. The supernatant was removed immediately and 3-ß hydroxyl steroid dehydrogenase (3ß-HSD) and 17ß-HSD were assayed using rat specific ELISA kits (Cusabio, USA)

#### Estimation of lipid peroxidation, non-enzymatic and enzymatic antioxidants

A part of the testis was homogenized using homogenizing buffer (10 times, w/v, 0.1 M phosphate buffer (pH=7.4)+150 mM KCl) to prepare 10% homogenate. A part of the homogenate was used for lipid peroxidation (LPO) and glutathione (GSH) estimations. The remaining part was centrifuged at 8500 g for 20 minutes in a refrigerated centrifuge to get supernatant (S) fraction. The ‘S’ fraction was used for measurement of superoxide dismutase (SOD), catalase (CAT), glutathione peroxidase (GPx), glutathione-S-transferase (GST) and glutathione reductase (GR) activities (5).

Briefly, LPO and GSH were estimated by the methods of Ohkawa et al. (16) and Elman (17), respectively. The activity of SOD was estimated by the method described by Kakkar et al. (18). CAT activity was estimated by the method described by Sinha (19). GPx, GST and GR activities were assayed by the methods of Rotruck et al. (20), Habig et al. (21) and Carlberg and Mannervik (22), respectively. Protein content in tissue homogenate was estimated by the method of Lowry et al. (23).

### Histological studies

The testicular tissues, previously kept in 10% formaldehyde were used for histological studies. The tissues were washed overnight in running water to remove remaining fixative. Dehydration was carried out to remove water using a series of gradually increasing concentrations of alcohol. These tissues were then cleared in xylol, embedded in wax and cut in sections of 5-µm thickness. The sections were recovered from wax blocks, stained with haematoxylin and eosin and analyzed by trinocular microscope with camera (24, 25).

### Statistical analysis

The results were expressed as mean ± SEM. Intergroup variations were evaluated by one way analysis of variance (ANOVA) followed by Dunnett’s test. Statistical significance was considered at P<0.05. The statistical analysis was performed using Graph Pad In Stat Software Inc., V. 3.06, San Diego, USA.

## Results

### Effects on weight of testes and epididymis

**Fig.1:**
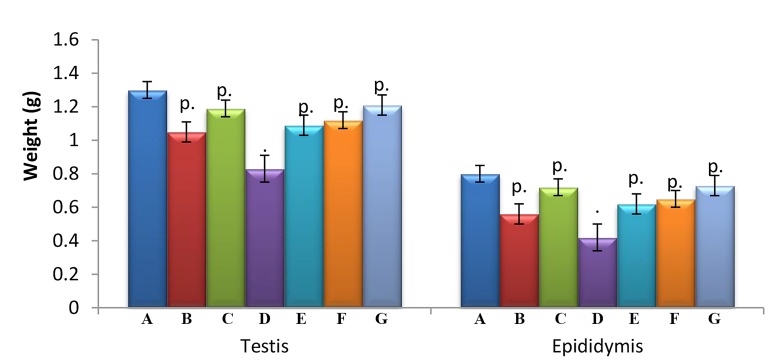
Effect of curcumin and quercetin in Cyp and Del-induced changes in sex organs weight. Each bar represents mean ± SEM of 6 rats. Cyp; Cypermethrin, Del; Deltamethrin, .; P>0.05, compared to group A, p.; Compared with group A and D both, A; Control, B; Cyp, C; Del, D; Cyp+Del, E; Cyp+Del+curcumin, F; Cyp+Del+quercetin, and G; Cyp+Del+curcumin+quercetin.

Non-significant decreases in weight of testis and epididymis were observed between exposure (B, C, and D) and treatment groups (E, F, and G) and control group A. The treatment groups E, F and G showed non-significant (P>0.05) increases in the weight of testes and epididymis as compared to exposure group D (Fig .1).

### Effects on sperm parameters

**Fig.2:**
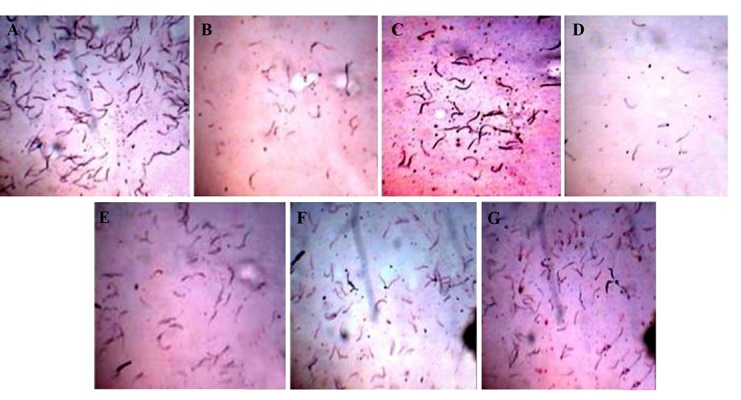
Effect of curcumin and quercetin on Cyp and Del-induced changes in testicular sperm counts. A. Control, B. Cyp, C. Del, D. Cyp+Del, E. Cyp+Del+curcumin, F. Cyp+Del+quercetin, and G. Cyp+Del+curcumin+quercetin. Cyp; Cypermethrin, Del; Deltamethrin.

Sperm head counts were significantly decreased in groups B, C, D, E, and F (29.38, 15.99, 40.46, 15.53 and 17.38%, respectively, P<0.01) and group G (5.15%, P>0.05) as compared to group A. Sperm motility was decreased significantly in groups B and (28.09 and 46.20%, respectively, P<0.01) and groups E and F (17.70 and 14.86%, respectively, P<0.05) whereas it decreased non-significantly (P>0.05) in groups C (14.69%) and G (2.09%) as compared to group A. On the other hand, in group E, F and G, we observed significant (P<0.01) increase in sperm head counts (41, 38.75 and 59.30%, respectively) and sperm motility (52.98, 58.26 and 82%, respectively) as compared to group D. Significant (P<0.01) increases in sperm abnormality were observed in groups B (61.37%), C (52.85%) and D (102.28%) as compared to control group A. Groups E, F and G showed significant increases in sperm abnormality (27.27% for group E (P<0.05), 22.73% for group F (P>0.05) and 8.43% for group G (P>0.05) as compared to group A. On the contrary, a significant (P<0.01) reduction in sperm abnormality was found in group E (37.07%), F (39.32%), and G (46.39%) as compared to group D (Table 1, Fig .2).

### Effects on testosterone, follicle stimulating hormone and luteinizing hormone

Testosterone levels decreased significantly in groups B, C, and D (73.37, 65.47, and 90.70%, respectively, P<0.01 for all groups) and groups E and F (54.99 and 56.47%, respectively, P<0.05 for all groups) and non- significantly (P>0.05) in group G (18.84%) as compared to group A. Significant (P<0.05) increases in testosterone was observed in groups E, F and G (p<0.01) when compared to group D. A decrease in FSH level was observed in groups B, C, E, F, and G (57.55, 47.08, 27.95, 29.06, and 16.77%, respectively, P>0.05 for all groups) and D (64.55%) as compared to group A. Groups E, F (p<0.05) and G (p<0.01) showed significant increases in FSH level as compared to group D. A significant decrease in LH level was observed in groups B, C, D, E, and F (56.76, 52.52,72.97,49.69 and 57.92%, respectively, p<0.01 or all groups) and G (37.37%, p<0.05) as compared to group A. Groups E, F (p<0.05) and G (p<0.01) showed significant increases in LH level as compared to group D (Table 2). 

### Effects on steroidogenic enzymes

Non-significant (P>0.05) decreases 3ß-HSD was observed in groups B (67.39%), C (49.34%), D (76.84%), E (17.28%), F (31.95%) and G (3.26%) as compared to group A, whereas significant (p<0.05) increases were found in groups E, F and G (p<0.01) as compared to group D. 17ß-HSD activity was significantly (p<0.01) decreased in groups B (58.65%), C (52.88%), D (80.28%) and F (52.88%) and also in groups E (44.23%, p<0.05) and G (5.76%, P>0.05) as compared to group A. Significant (P>0.05) increases in 17ß-HSD activity were observed in groups E, F and G (p<0.01) as compared to group D (Table 3).

**Table 1:** Effect of curcumin and quercetin on Cyp and Del-induced changes in sperm parameters


Parameter	Group A	Group B	Group C	Group D	Group E	Group F	Group G

Sperm count×10^6^/g tissue	72.22 ± 1.176	51.00 ± 0.730q^**^	60.666 ± 2.028r^**^	43.00 ± 1.751^**^	61 ± 2.129r^**^	59.666 ± 2.275r^**^	68.5 ± 2.487r^•^
Sperm motility (%)	70.475 ± 3.367	50.675 ± 2.54q^**^	60.12 ± 3.245r^•^	37.911 ± 2.909^**^	60.00 ± 2.745r^*^	58.00 ± 2.160r^*^	69.00 ± 1.506r^•^
Sperm abnormality (%)	43.998 ± 3.246	71.00 ± 1.807r^**^	67.251 ± 2.627r^**^	89.00 + 3.183^**^	56.00 ± 3.724r^*^	54.00 ± 2.769r^•^	47.71 ± 2.911r^•^


Data are presented as mean ± SEM of 6 rats in each group. Cyp; Cypermethrin, Del; Deltamethrin, •; P>0.05, *; P<0.05, **; P<0.01 compared with group A, p; P>0.05, q; P<0.05, r; P<0.01 compared with group D, Group A; Control, B; Cyp, C; Del, D; Cyp+Del, E; Cyp+Del+curcumin, F; Cyp+Del+quercetin, and G; Cyp+Del+curcumin+quercetin.

**Table 2:** Effect of curcumin and quercetin on Cyp and Del-induced changes in sex hormone level


Parameter	Group A	Group B	Group C	Group D	Group E	Group F	Group G

Testosterone (ng/ml)	3.635 ± 0.745	0.968 ± 0.302p^**^	1.255 ± 0.170p^**^	0.338 ± 0.137^**^	1.636 ± 0.492q^*^	1.585 ± 0.495q^*^	2.95 ± 0.556r^•^
FSH (mIU/ml)	2.415 ± 0.446	1.025 ± 0.112p^•^	1.278 ± 0.169p^•^	0.856 ± 0.213^*^	1.74 ± 0.494q^•^	1.713 ± 0.492q^•^	2.01 ± 0.454r^•^
LH (mIU/ml)	1.98 ± 0.325	0.856 ± 0.213p^**^	0.94 ± 0.220p^**^	0.535 ± 0.126^**^	0.996 ± 0.118q^**^	0.833 ± 0.113q^**^	1.24 ± 0.112r^*^


Data are presented as mean ± SEM of 6 rats in each group. Cyp; Cypermethrin, Del; Deltamethrin, •; P>0.05, *; P<0.05, **; P<0.01 compared with group A, p; P>0.05, q; P<0.05, r; P<0.01 compared with group D, Group A; Control, B; Cyp, C; Del, D; Cyp+Del, E; Cyp+Del+curcumin, F; Cyp+Del+quercetin, and G; Cyp+Del+curcumin+quercetin.

**Table 3:** Effect of quercetin and curcumin on Cyp and Del-induced changes in steroidogenic enzymes


Parameter	Group A	Group B	Group C	Group D	Group E	Group F	Group G

3-β HSD (pg/ml)	0.92 ± 0.283	0.30 ± 0.125p^•^	0.466 ± 0.105p^•^	0.213 ± 0.106^•^	0.761 ± 0.213q^•^	0.626 ± 0.175q^•^	0.89 ± 0.274r^•^
17-β HSD (ng/ml)	1.04 ± 0.112	0.43 ± 0.104p^**^	0.49 ± 0.105p^**^	0.205 ± 0.095^**^	0.58 ± 0.124q^*^	0.49 ± 0.100q^**^	0.98 ± 0.113r•


Data are presented as mean ± SEM of 6 rats in each group. Cyp; Cypermethrin, Del; Deltamethrin, •; P>0.05, *; P<0.05, **; P<0.01 compared with group A, p; P>0.05, q; P<0.05, r; P<0.01 compared with group D, Group A; Control, B; Cyp, C; Del, D; Cyp+Del, E; Cyp+Del+curcumin, F; Cyp+Del+quercetin, and G; Cyp+Del+curcumin+quercetin.

### Effects on lipid peroxidation, and non-enzymatic and enzymatic antioxidants

Significant (p<0.01) increases in LPO level were observed in groups B (142.65%), C (80%), D (188.94%), E (144.70%), F (127.48%) and G (47.70%) as compared to group A. Significant (p<0.05) decreases in LPO level were observed in groups E (15.52), F (86.87%) and also in group G (48.87%, p<0.01) as compared to group D. GSH level was significantly (p<0.01) decreased in group B (40%), C (31.98%), D (75.82%), E (54.69%) and F (46.36%) and non-significantly (P>0.05) in group G (6.67%) as compared to group A. Groups E (87.39%, p<0.05), F(121.81%, p<0.01) and G (285.97%, p<0.01) showed significant increases in GSH level compared to group D (Table 4). The results of SOD, CAT, GPx, GR and GST activities are summarized in Table 4. Rats in groups B, C, D, E, and F showed significant (p<0.01) decreases (44.69, 28.36, 66.68, 39.03 and 31.91%, respectively) in SOD activity, compared to group A, whereas a non-significant (9.51%, P>0.05) decrease was observed in group G. On the other hand, significant (p<0.01) increases in SOD level were observed in groups E (82.98%), F (104.36%) and G (171.60%) when compared to group D. CAT activity was significantly decreased in groups B, C and D (35.77, 18.26 and 50.71%, respectively, p<0.01 for all groups), E and F (6.23 and 6.76%, respectively, p<0.05 for both groups) and non significant in group G (2.88%, P>0.05) as compared to group A.

However, significant (p<0.05) increases were observed in groups E (89.18%), F (90.27%), and G (97%) when compared to group D. Significant (p<0.05) decreases in GPx activity were observed in groups B (36.32%), D (55.59%, p<0.01) and non-significant (P>0.05) decreases were found in groups C (21.23%), F (22.21%), E (27.54%) and G (3.84%) as compared to group A. Significant (p<0.05) increases in GPx activity were observed in groups E (63.17%), F (75.18%) and G (116.55%, p<0.01) as compared to group D. GR activity was significantly (p<0.05) reduced in groups B (31.94%), E (27.77%), F (25.83%) and D (61.55%, p<0.01) and non-significantly (P>0.05) in groups C (20.96%) and G (6.38%) as compared to group A.

On the other hand, significant (p<0.01) increases were observed in groups E (87.84%), F (92.89%) and G (143.48%) as compared to group D. The GST activity was significantly (p<0.01) decreased in groups B (7.19%), D (17.62%), E (14.13%), F (14.40%), G (8.76%) and C (4.60%, p<0.05) as compared to group A. Significant increases in GST were recorded in groups E and F (4.24, 3.91%, respectively, p<0.05 for both groups) and group G 10.75%, p<0.01, as compared to group D (Table 4).

**Table 4:** Effect of curcumin and quercetin on Cyp and Del-induced changes in lipid peroxidation, non- enzymatic and enzymatic antioxidants


Parameter	Group A	Group B	Group C	Group D	Group E	Group F	Group G

LPO (nmoles MDA/hours/gtissue)	3.165 ± 0.2749	7.68 ± 0.166q^**^	5.726 ± 0.148r^**^	9.145 ± 0.489^**^	7.725 ± 0.319q^* *^	7.2 ± 0.402r^**^	4.675 ± 0.309r^*^
GSH(µmole/g tissue)	2.920 ± 0.288	1.735 ± 1.145r^**^	1.986 ± 0.224r^**^	0.706 ± 0.071^**^	1.566 ± 0.03827r^**^	1.323 ± 0.0388q^**^	2.725 ± 0.1385r^•^
SOD (nmole/minutes/mg protein)	31.911 ± 1.262	17.648 ± 1.631r^**^	22.858 ± 1.474r^**^	10.631 ± 0.5518^**^	21.726 ± 0.9145r^**^	19.453 ± 1.170r^**^	28.874 ± 0.7624r^•^
CAT(µ mole/minutes/mg protein)	74.14 ± 1.193	47.62 ± 1.335r^**^	60.596 ± 1.295r^**^	36.538 ± 1.331^**^	69.521 ± 0.659r^*^	69.123 ± 0.620r^*^	72.00 ± 0.7857r^•^
GPx (nmole/minutes/mg protein)	8.396 ± 1.005	5.346 ± 0.715q^*^	6.613 ± 0.774q^•^	3.728 ± 0.760^**^	6.531 ± 0.771q^•^	6.083 ± 0.736q^•^	8.073 ± 0.719r^•^
GR (nmole/minutes/mg	3.553 ± 0.339	2.418 ± 0.250q^*^	2.808 ± 0.2778r^•^	1.366 ± 0.1686^**^	2.635 ± 0.185r^*^	2.566 ± 0.2008r^*^	3.326 ± 0.1578r^•^
GST (µmole/minutes/mg protein)	91.015 ± 0.681	84.471 ± 1.141r^**^	86.823 ± 0.775r^*^	74.973 ± 1.284^**^	78.153 ± 0.673p^**^	77.906 ± 0.741p^**^	83.038 ± 0.791r^**^


Data are presented as mean ± SEM of 6 rats in each group. Cyp; Cypermethrin, Del; Deltamethrin, •; P>0.05, *; P<0.05, **; P<0.01 compared with group A, p; P>0.05, q; P<0.05, r; P<0.01 compared with group D, Group A; Control, B; Cyp, C; Del, D; Cyp+Del, E; Cyp+Del+curcumin, F; Cyp+Del+quercetin, and G; Cyp+Del+curcumin+quercetin.

**Fig.3:**
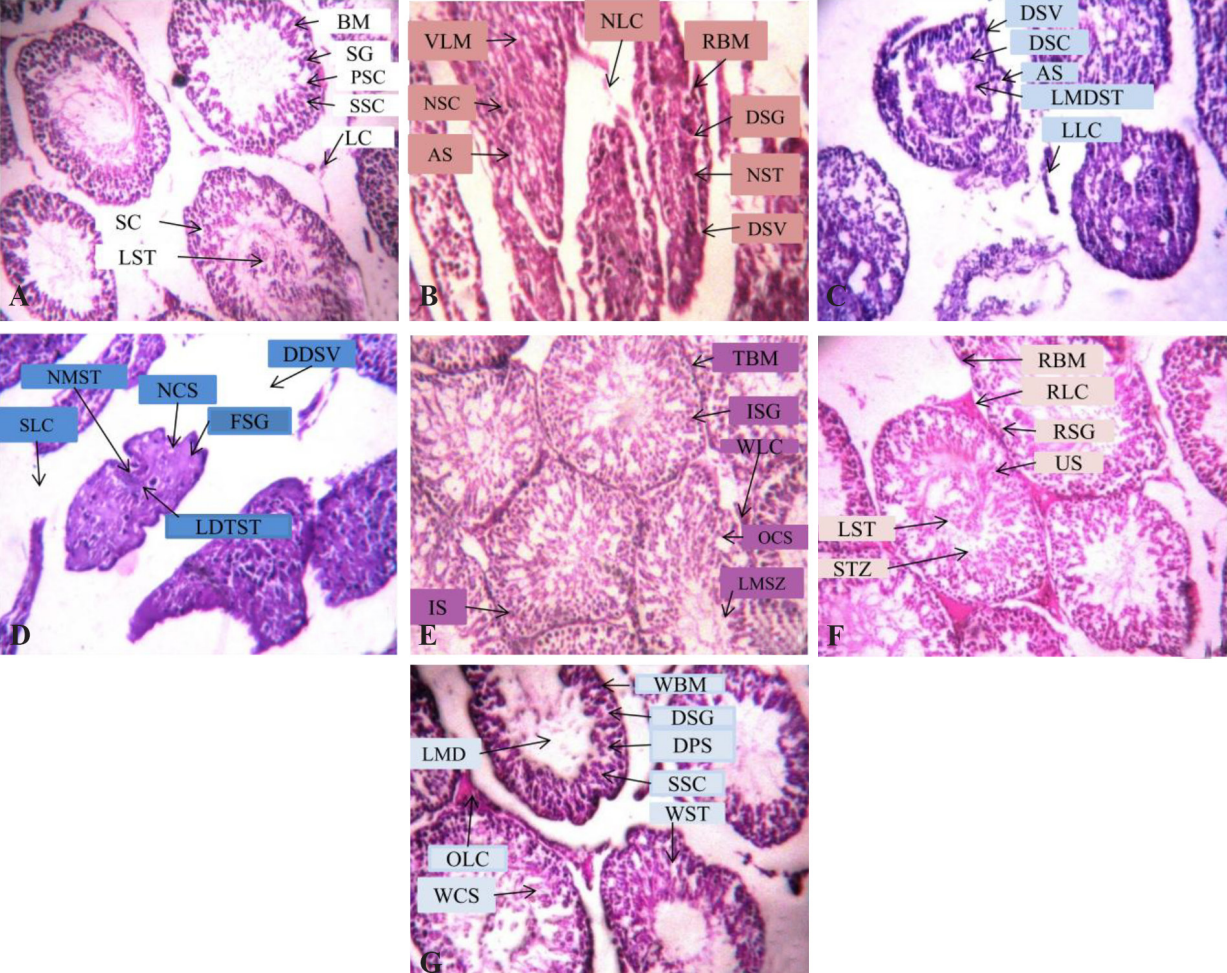
Effect of curcumin and quercetin on Cyp and Del-induced changes in testicular sperm counts. A. Control, B. Cyp, C. Del, D. Cyp+Del, E. Cyp+Del+curcumin, F. Cyp+Del+quercetin, and G. Cyp+Del+curcumin+quercetin. Cyp; Cypermethrin, Del; Deltamethrin, LC; Leydig cells, BM; Basement membrane, SG; Spermatogonia, PSC; Primary spermatocyte, SSC; Secondary spermatocyte, LST; Lumen filled with spermatids, SC; Sertoli cells, RBM; Ruptured basement membrane, DSV; Disorganized structure of seminal vesicles, NSC; Necrosis of sertoli cells, VLM; Vacuolation of lumen, NLC; Necrosis of Leydig cells, AS; Arrested stages of spermatogenesis, DSG; Disorganized spermatogonia, NST, Necrosis of spermatids, DSV; Disorganization of seminal vesicles, LLC; Loosed Leydig cells, DSC; Degeneration of sertoli cells, AS; Arrested spermatogenesis, LMDST; Lumen filled with dead spermatids, DDSV; Disorganization and disappearance of seminal vesicles, NSC; Necrosis of sertoli cell, FSG; Fading of spermatogonia, NMST non-motile spermatids, SLC; Scattering of Leydig cells, LDTST; Lumen filled with dead and tailless spermatids, TBM; Thick basement membrane, ISG; Increased spermatogonia, WLC; Well-developed Leydig cells, IS; Increased spermatogenesis, OCS; Organized sertoli cells, LMSZ; Lumen filled with spermatozoa, RBM; Recovery of basement membrane, RLC; Recovery of Leydig cell, RSG; Recovery of spermatgonia, US; Unaffected spermatogenesis, LST; Lumen filled with spermatids, SZT; Spermatozoa with tail, WBM; Well-shaped basement membrane, DSG; Dense structured spermatogonia, DPS; Densely packed primary spermatocyte, SSC; Secondary spermatocyte, WST; Well-developed spermatids with tails, LMD; Lumen filled with dense materials, OLC; Organized Leydig cells, and WCS; Well-shaped sertoli cells.

### Histology of testes

The histology of testes of control rats showed seminiferous tubules separated by basement membrane containing Leydig cells. The germinal epithelium consisted of concentric layers of germs cells viz. spermatogonia, primary and secondary spermatocytes, lumen filled with spermatids and spermatozoa with tail and sertoli cells (Fig .3A). The testes of rats treated with single and combined exposure to Cyp and Del, showed ruptured basement membrane, disorganized seminal vesicles, necrosis of Leydig cells and sertoli cells, vacuolation of lumen, arrested stages of spermatogenesis including disorganized spermatogonia, increased intertubular space and lumen with cellular debris (Fig .3B-D). Treatment with curcumin, quercetin and combination of both recovered the histological damage induced by the insecticides (Fig .3E-G).

## Discussion

Cyp and Del showed toxic effects in reproductive system of male Wistar rats. The reproductive toxicity caused by these insecticides was ameliorated by curcumin and quercetin. The weight of the testes and epididymis decreased following single as well as combined exposure to Cyp and Del, as compared to the control. The male reproductive toxicity of Cyp (25) and Del (26) has been previously reported in laboratory animals. The decrease in testes and epididymis weight observed in the present study may be due to the direct cytotoxic action of these insecticides on testicular tissue. Also, a significant decrease in testicular sperm head counts was observed following single and combined exposure to Cyp and Del as compared to the control. Probably, accumulation of the insecticides in the testicular tissue may have adversely affected the sertoli cell population leading to compromised spermatogenesis and reduction in sperm head counts. Decreases in serum testosterone which were observed in the present experiment and reported by previous studies, may also be responsible for the reduction in sperm head counts (27). Also, an increase in ROS has been reported to decrease sperm counts (28). In the present study, we observed increases in LPO and decreases in enzymatic and non-enzymatic antioxidants following exposure to Cyp and Del. So, decreased sperm counts may also be due to increases in lipid peroxidation and excessive generation of ROS. Dichlorvos (29) has been reported to increase sperm abnormality and reduce sperm motility. We also observed increases in sperm abnormality and reductions in sperm motility after Cyp and Del exposure in our study, which were possibly due to excessive ROS production and decreases in testosterone level.

The endocrine disruptive action of Del (30) and Cyp (31) has been previously reported, though the mechanism is largely unknown. The decrease in testosterone level following Cyp and Del exposure observed in this study, may be due to the direct effect of these pyrethroids on the androgen biosynthesis pathways in the testes or alterations in gonadotropins levels. We observed reduction in steroidogenic enzymes (i.e. 3ß-HSD and 17-ßHSD) after exposure to Cyp and Del. As there was marked increases in oxidative stress in the testicular tissue, this reduction in 3ß-HSD and 17ß-HSD may either be due to Leydig cell damage or direct action of these insecticides on gene expression of 3ß-HSD and 17ß-HSD.

StAR protein transports cholesterol from the cytoplasm to the mitochondrial matrix. The transport of cholesterol is a rate-limiting step in testosterone biosynthesis. There are reports that pyrethroids reduce StAR protein expression (32). Hence, decreases in testosterone observed in the present study following exposure to Cyp and Del, may be possibly induced by inhibition of StAR protein expression. Spermatogenesis is also controlled by the gonadotropins and any alteration in level of gonadotropins may impair spermatogenic activity. Hu et al. (33) reported decreased testosterone after Cyp exposure with concomitant increases in level of FSH and LH, possibly due to negative feedback inhibition. On the other hand, Issam et al. (34) reported decreases in FSH, LH and testosterone after Del exposure in 45 and 60-day experiments. In our study, we observed decreased in testosterone, FSH and LH levels following Cyp and Del exposure. It is possible that the effect of these pyrethroids on pituitary gonadotropin hormones and testicular hormones, is dependent on time of exposure and testicular tissue being their primary target. During short-term exposure, due to direct cytotoxic effect of Cyp and Del on testicular tissue, a decrease in testosterone is observed, which in turn increases the level of FSH and LH due to negative feedback inhibition. However, during long- term exposure, it is possible that the anterior pituitary is also affected by Cyp and Del along with testes, which may result in decreases in testosterone, FSH and LH levels, as observed in present study.

Increased lipid peroxidation impairs membrane functions by decreasing membrane fluidity and changing the activity of membrane-bound enzymes and receptors. Lipid peroxidation products (lipid radical and lipid peroxide) are harmful to the cells and are associated with a number of pathological conditions. In the present study, significant increases in the level of LPO was observed following single and combined exposure to Cyp and Del as compared to control group A. Increased LPO has been reported after Cyp exposure in rats brain and liver (35). We observed significant decreases in GSH level after single and combined exposure of Cyp and Del as compared to the control. The decrease in GSH may be due to increased utilization of GSH for detoxification of excessive free radicals generated after pesticide exposure.

Superoxide dismutase is the first line of defense against deleterious effects of oxygen radicals in the cell. It acts by catalyzing the dis-mutation of superoxide radicals to hydrogen peroxide and molecular oxygen. Significant decreases in the SOD activity were observed in the present study, which may be due to the decrease in the ability of the tissues to handle extra free radical. These extra free radicals may attack the thiol group of cysteine residues of proteins and polysaturated fatty acids of biological membranes. Catalase is present ubiquitously in nearly all living organisms exposed to oxygen and catalyzes the decomposition of hydrogen peroxide to water and oxygen (36). In our study, a significant decrease in CAT activity was observed, possibly because of inactivation of the enzyme by excessive ROS production. Also, decreased CAT activity was observed by Latchoumycandane and Mathur following methoxychlorexposure in rats (37).

The present study showed significant decreases in GPx activity which may be due to reduced level of GSH, a substrate of GPx. GR is a member of the pyridine-nucleotide disulfide oxidoreductase family of flavo enzymes which catalyzes the reduction of glutathione disulfide (GSSG) to its reduced form GSH, in the presence of NADPH. The level of GR was decreased in this study, possibly due to the damage caused by Cyp and Del to the tertiary structure of the enzyme. GST catalyzes the conjugation of GSH to electrophiles and protects cellular components from oxidative damage (38). In the present study, significant decreases in the GST activity were observed. Decreased GST may be due to the affinity of this enzyme to the hydrophobic compounds like pyrethroid insecticides. Decreased GST has been previously reported following exposure to phosphorothionate (39) in male rats.

Additionally, exposure to Cyp and Del resulted in marked histo-architectural disturbances in testis. These changes were possibly caused by ROS-induced cell damage. The damage in sperm mother cells as well as supporting sertoli cells resulted in gross changes in sperm parameters and decrease in testosterone.

Curcumin and quercetin are phytochemicals with proven antioxidant and cyto-protective activities. Treatment of Cyp and Del-exposed rats with curcumin and quercetin in the present study, increased sex organs weights, sperm count, sperm motility, sex hormones (testosterone, LH and FSH) levels, and steroidogenic enzymes (3ß-HSD and 17ß-HSD) and decreased sperm abnormalities. Treatment with curcumin and quercetin also restored Cyp and Del-induced histo-architectural disturbances by their antioxidant and cyto-protective activities. Since we observed direct cytoprotective effect of these antioxidants, particularly the restoration of testicular histo-architecture, it is possible that curcumin and quercetin have crossed the blood-testes-barrier. The increase in antioxidant defense of the testis, as reflected by increased GSH, CAT, SOD, GPx, GST and GR activities and decreased LPO levels, indicated curcumin and quercetin-mediated scavenging of the hydroxyl, peroxy, and superoxide radicals.

Moreover, increased antioxidant defense protected sertoli and Leydigcells with concomitant increases in the level of sex hormones viz. testosterone, FSH and LH. There are reports of feedback regulation of testosterone biosynthesis by FSH and LH. Hu et al. (33) reported decreases in testosterone and increases in FSH and LH level following Cyp exposure. In our study, we observed increases in all sex hormones. Enhanced testosterone level may by cyto-protective effect of curcumin and quercitin on testicular tissue. Since the animals were orally fed with curcumin and quercitin, the effect of these phytochemicals on other systems can not be ruled out. We propose that these phytochemicals may also protect the pituitary gland and enhance the level of FSH and LH. We also observed increases in steroidogenic enzymes 3ß-HSD and 17ß-HSD, responsible for enhanced biosynthesis of testosterone. Although, no data is available on the mechanism of induction of steroidogenic enzymes by natural antioxidants, it is possible that curcumin and quercetin might have up-regulatedthe gene expression of these enzymes.

## Conclusion

This study indicated that the combined exposure to Cyp and Del was more toxic than exposure to each of the insecticide alone. The 45-day exposure to Cyp and Del showed marked decreases in sperm motility and sperm head counts, increases in sperm abnormality and decreases in testosterone, FSH, LH, 3ß-HSD and 17ß-HSD in serum. Enhanced activities of steroidogenic enzymes (3ß-HSD and 17ß-HSD) and concomitant increased levels of testosterone were mainly responsible for ameliorating effect of curcumin and quercetin. We also observed decreases in enzymatic and non-enzymatic antioxidants and disturbance in testicular histo-architecture in the exposed rats. Treatment with curcumin and quercetin ameliorated Cyp and Del-induced toxicity by improving the reproductive system. Curcumin showed slightly better activity as compared to quercetin. Our study further showed that combined treatment of curcumin and quercetin possesses higher activity as compared to treatment with each one alone. 

## References

[1] Casida JE, Quistad GB (1998). Golden age of insecticide research: past, present, or future?. Annu Rev Entomol.

[2] Saillenfait AM, Ndiaye D, Sabaté JP (2015). Pyrethroids: exposure and health effects-an update. Int J Hyg Environ Health.

[3] Tsuji R, Yamada T, Kawamura S (2012). Mammal toxicology of synthetic pyrethroids. Top Curr Chem.

[4] Sharma P, Huq AU, Singh R (2014). Cypermethrin-induced reproductive toxicity in rat is prevented by resveratrol. J Hum Reprod Sci.

[5] Sharma P, Singh R, Jan M (2014). Dose dependent effect of deltamethrin in testis, liver and kidney of Wistar rats. Toxicol Int.

[6] Luthra PM, Singh R, Chandra R (2001). Therapeutic uses of Curcuma longa (turmeric). Indian J Clin Biochem.

[7] D'Andrea G (2015). Quercetin: a flavonol with multifaceted therapeutic applications?. Fitoterapia.

[8] Lonare M, Kumar M, Raut S, More A, Doltade S, Badgujar P (2016). Evaluation of ameliorative effect of curcumin on imidacloprid-induced male reproductive toxicity in Wistar rats. Environ Toxicol.

[9] Farombi EO, Abarikwu SO, Adesiyan AC, Oyejola TO (2013). Quercetin exacerbates the effects of subacute treatment of atrazine on reproductive tissue antioxidant defencesystem, lipid peroxidation and sperm quality in rats. Andrologia.

[10] Ogawa M, Maruyama S, Ostsubo T, Tsuda S, Tsuji JK (1991). Low toxic emulsifiable concentrates of pyrethroids with polyethylene glycol and polypropylene glycol. J Pesticide Sci.

[11] Singh R, Sharma P (2011). Hepatoprotective effect of Curcumin on Lindane induced oxidative stress in male wistar rats. Toxicol Int.

[12] Zargar S, Siddiqi NJ, Al Daihan SK, Wani TA (2015). Protective effects of quercetin on cadmium fluoride induced oxidative stress at different intervals of time in mouse liver. Acta Biochim Pol.

[13] Sharma P, Khan I, Singh R (2016). Efficacy of Curculigoorchioides in deltamethrin induced reproductive system impairment in male Wistar rats. Asian J Pharmaceutics.

[14] Choi EK, Tsunekawa N, Kanai Y, Kurohmaru M (2008). A new preparation protocol for measurement of testicular sperm production. J Reprod Dev.

[15] Williams J, Gladen BC, Schrader SM, Turner TW, Phelps JL, Chapin RE (1990). Semen analysis and fertility assessment in rabbits: statistical power and design considerations for toxicology studies. Fund Appl Toxicol.

[16] Ohkawa H, Ohishi N, Yagi K (1979). Assay for lipid peroxides in animal tissues by thiobarbituric acid reaction. Anal Biochem.

[17] Ellman GL (1959). Tissue sulfhydryl groups. Arch Biochem Biophys.

[18] Kakkar P, Das B, Viswnathan PN (1984). A modified spectrophotometric assay of superoxide dismutase. Indian J Biochem Biophys.

[19] Sinha AK (1972). Colorimetric assay of catalase. Anal Biochem.

[20] Rotruck JT, Pope AL, Ganther HE, Swanson AB, Hafeman DG, Hoekstra WG (1973). Selenium: Biochemical role as a component of glutathione peroxidase. Science.

[21] Habig WJ, Pabst MJ, Jakoby WB (1974). Glutathione S transferases.The first enzymatic step in mercapturic acid formation. J Biol Chem.

[22] Carlberg I, Mannervik B (1985). Glutathione reductase. Methods Enzymol.

[23] Lowry OH, Rosebrough NJ, Farr AL, Randall RJ (1951). Protein measurement with the Folin phenol reagent. J Biol Chem.

[24] Culling CF (1949). The mass staining of paraffin sections before the removal of wax. J Clin Pathol.

[25] Ikpeme EV, Okono LE, Udensi OU (2016). Detrimental effect of chlorpyrifos and cypermethrin on reproductive physiology of male albino rats. Res J Environ Toxicol.

[26] Saillenfait AM, Ndiaye D, Sabaté JP, Denis F, Antoine G, Robert A (2016). Evaluation of the effect of deltamethrin on fetal rat testis. J Appl Toxicol.

[27] Liu L, Hu JX, Wang H, Chen BJ, He Z, Xu LC (2010). Effects of beta-cypermethrin on male rat reproductive system. Environ Toxicol Pharmacol.

[28] Hipler UC, Gِrnig M, Hipler B, Rِmer W, Schreiber G (2000). Stimulation and scavestrogen-induced inhibition of reactive oxygen species generated by rat sertoli cells. Arch Androl.

[29] Okamura A, Kamijima M, Shibata E, Ohtani K, Takagi K, Ueyama J (2005). A comprehensive evaluation of the testicular toxicity of dichlorovos in Wistar rats. Toxicology.

[30] Ben Slima A, Chtourou Y, Barkallah M, Fetoui H, Boudawara T, Gdoura R (2016). Endocrine disrupting potential and reproductive dysfunction in male mice exposed to deltamethrin. Hum Exp Toxicol.

[31] Jin Y, Wang L, Ruan M, Liu J, Yang Y, Zhou C (2011). Cypermethrin exposure during puberty induces oxidative stress and endocrine disruption in male mice. Chemosphere.

[32] Wang H, Wang Q, Zhao XF, Liu P, Meng XH, Yu T (2010). Cypermethrin exposure during puberty disrupts testosterone synthesis via downregulation StAR in mouse testes. Arch Toxicol.

[33] Hu JX, Li YF, Li J, Pan C, He Z, Dong HY (2013). Toxic effects of cypermethrin on the male reproductive system: with emphasis on the androgen receptor. J Appl Toxicol.

[34] Issam C, Samir H, Zohra H, Monia Z, Hassen BC (2009). Toxic response to Deltamethrin (DM) low doses on gonads, sex hormones and lipid peroxidation in male rats following subcutaneous treatment. J Toxicol Sci.

[35] Giray B, Gurbay A, Hincal F (2001). Cypermethrin-induced oxidative stress in rat brain and liver is prevented by Vitamin E or Allopurinol. Toxicol Lett.

[36] Chelikan P, Fita I, Loewen PC (2004). Diversity of structures and properties among catalases. Cell Mol Life Sci.

[37] Latchoumycandane C, Mathur PP (2002). Induction of oxidative stress in the rat testis after short-term exposure to the organochlorine pesticide methoxychlor. Arch Toxicol.

[38] Hayes JD, Pulford DJ (1995). The glutathione S-transferase supergene family: regulation of GST and the contribution of the isoenzymes to cancer chemoprotection and drug resistance. Crit Rev Biochem Mol Biol.

[39] Khan IA, Reddy BV, Mahboob M, Rahman MF, Jamil K (2001). Effects of phosphorothionate on the reproductive system of male rats. J Environ Sci Health B.

